# Investigation of Plasmid-Mediated Colistin Resistance Genes (mcr-1–8) in Enterobacterales Isolates

**DOI:** 10.7759/cureus.61538

**Published:** 2024-06-02

**Authors:** Melahat Gürbüz, Emek Türkekul Şen, Merih Şimşek, Cengiz Demir

**Affiliations:** 1 Department of Medical Microbiology, Afyonkarahisar Health Sciences University, Afyonkarahisar, TUR; 2 Microbiology Clinic, Ankara Training and Research Hospital, Ankara, TUR; 3 Department of Nutrition and Dietetics, Afyonkarahisar Health Sciences University, Afyonkarahisar, TUR

**Keywords:** multiplex pcr, mcr gene, broth microdilution, colistin, klebsiella pneumoniae, escherichia coli, carbapenem-resistant enterobacterales

## Abstract

Background

The escalating global rise in multidrug-resistant gram-negative bacteria presents an increasingly substantial threat to patient safety. Over the past decade, carbapenem-resistant Enterobacterales (CRE) have emerged as one of the most critical pathogens in hospital-acquired infections, notably within intensive care units. Colistin has become one of the last-resort antimicrobial agents utilized to combat infections caused by CRE. However, the use of colistin has been accompanied by a notable increase in the prevalence of colistin-resistant bacteria. This study aimed to investigate plasmid-mediated colistin resistance genes ranging from *mcr*-1 to *mcr*-8 among members of the Enterobacterales order.

Materials and methods

This prospective study was conducted in the microbiology laboratory of Afyonkarahisar Health Sciences University Health Research and Practice Center between May 1, 2021 and July 31, 2022. A total of 2,646 Enterobacterales isolates were obtained from all culture-positive clinical samples sent from various clinics. Of these, 79 isolates exhibiting resistance to carbapenem antibiotics were included in the study. Among the 79 isolates, the presence of *mcr*-1 to *mcr*-8 genes was investigated in 27 isolates that were shown to be resistant to colistin. The identification of bacteria at the species level and antibiotic susceptibility tests were conducted using the VITEK 2 automated system (bioMérieux, USA). Colistin resistance among Enterobacterales strains exhibiting carbapenem resistance was evaluated using the broth microdilution technique (ComASP™ Colistin, Liofilchem, Italy), in accordance with the manufacturer's instructions.

Results

In our in vitro investigations, the minimum inhibitory concentration (MIC) values for meropenem were determined to be >8 µg/ml, whereas for colistin, the MIC50 value was >16 µg/ml and the MIC90 value was 8 µg/ml. A total of 27 colistin-resistant strains were identified among the 79 carbapenem-resistant Enterobacterales strains analyzed. The most prevalent agent among colistin-resistant strains was *Klebsiella pneumoniae *(*K.* *pneumoniae*), representing 66.7% of the isolates. This was followed by *Proteus mirabilis *(*P.* *mirabilis*) with 29.6% and *Escherichia coli *(*E. coli*) with 3.7%. The colistin resistance rate among carbapenem-resistant strains was found to be 34.2%, with colistin MIC values in strains tested by the broth microdilution method ranging from 4 to >16 µg/ml concentrations. In polymerase chain reaction (PCR) studies, the *mcr*-1 gene region was successfully detected by real-time PCR in the positive control isolate. Nevertheless, none of the gene regions from *mcr*-1 to *mcr*-8 were identified in our study investigating the presence of plasmid-mediated genes using a multiplex PCR kit.

Conclusion

Although our study demonstrated the presence of increased colistin resistance rates in carbapenem-resistant Enterobacterales isolates, it resulted in the failure to detect genes from *mcr*-1 to *mcr*-8 by the multiplex PCR method. Therefore, it is concluded that the colistin resistance observed in Enterobacteriaceae isolates in our region is not due to the *mcr* genes screened, but to different resistance development mechanisms.

## Introduction

The significant increase in multidrug-resistant (MDR) microorganisms today, particularly the rapid rise of gram-negative bacteria resistant to the carbapenem group of antibiotics, has led to the reintroduction and last-resort clinical use of some older antimicrobials that had previously fallen out of use. However, the emergence of a new plasmid-mediated colistin resistance gene, named *mcr*-1, in November 2015, has raised concerns about the clinical efficacy of colistin. Since its initial identification, the *mcr-1* gene has been detected in a variety of bacterial isolates belonging to the Enterobacterales, including those recovered from humans, animal food products, and environmental samples. This resistance gene has spread to over 40 countries across five continents. Since the discovery of the first *mcr-1* gene, nine more *mcr *gene variants (*mcr-2 *to *mcr-10*) have been identified [1].

Colistin resistance is primarily attributed to phosphoethanolamine (pEA) transferases, which catalyze the addition of phosphoethanolamine (pEtN) to lipid A, thereby reducing the electrostatic attraction between colistin and the gram negative outer membrane [2]. On the other hand, the detection of *mcr-9* in colistin-sensitive strains has revealed that strains carrying *mcr* genes might not phenotypically exhibit colistin resistance due to low levels of gene expression [3].

Plasmids carrying the *mcr-1* gene not only have the ability to conjugate and transfer but also enhance the persistence of colistin resistance. To date, the *mcr-1* gene and its variants have been reported in many countries, with a total of 113 variants identified within 10 families of the *mcr* gene [4].

These plasmid-mediated resistances can quickly spread cross-species through horizontal gene transfer, leading to the emergence of MDR superbugs, which poses a significant challenge for clinicians [5]. The coexistence of mcr genes with other drug-resistant genes (*bla*NDM-13, *bla*TEM, *bla*SHV, tetA, floR, aac-3-IV, aadA1, fosA, and aac(6_)-lb) increases the likelihood of the emergence of drug-resistant super microorganisms [6,7]. The prevalence of multidrug resistance leads to an increase in hospital-acquired infections with limited treatment options, longer hospital stays, higher mortality rates, and increased costs [8].

In addition, it has been recently reported that the coexistence of *mcr-1* and carbapenemase genes has increased following the clinical reintroduction of colistin [9]. Therefore, there is an urgent need to develop new strategies to eradicate the spread of mcr genes and prolong the use of colistin as a last-resort antibiotic against carbapenem-resistant bacteria carrying the mcr gene. This study aims to investigate the plasmid-mediated resistance genes from *mcr-1 *to *mcr-8 *in members of the Enterobacterales.

## Materials and methods

This prospective observational study was conducted between May 1, 2021 and July 31, 2022 in the microbiology laboratory of the Afyonkarahisar Health Sciences University Health Research and Practice Center. The hospital is a tertiary university hospital located in western Turkey with a total of 655 beds, including 103 intensive care unit beds in three levels of acuity. The study was conducted with the approval of the Clinical Research Ethics Committee at Afyonkarahisar Health Sciences University (Decision: 2021/227).

Collection of bacterial strains

A total of 2,646 Enterobacterales isolates were identified from all clinical samples sent to the laboratory during the study period. Of these, 79 isolates that were determined to be resistant to carbapenem group antibiotics were included in the study. Of 79 isolates, 27 were resistant to colistin. The presence of genes from *mcr-1 *to* mcr-8* was investigated in these strains.

Identification of bacterial strains and antibiotic susceptibility testing

Clinical samples such as blood, cerebrospinal fluid, urine, sputum, and wound swabs sent to the laboratory were cultured on blood agar, chocolate agar, and EMB agar media and incubated at 37°C for 24 hours. After incubation, pure cultures of the bacteria were obtained by subculturing. Species-level identification of the bacteria and antibiotic susceptibility testing were performed using the VITEK 2 automated system (bioMérieux, USA). Resistant strains were confirmed using the gradient diffusion test method. Standard strains *E. coli *ATCC 25922, *E. coli* NTCC 13846 (resistant), and *K. pneumoniae *ATCC 700603 were used in all phenotypic susceptibility tests. Strains were stored in tryptic soy broth with 15% glycerol at -80°C until molecular testing was performed. Antibiogram results were evaluated according to the recommendations of the European Committee on Antimicrobial Susceptibility Testing (EUCAST) [10].

Determination of colistin resistance by the broth microdilution method

Colistin resistance in Enterobacterales strains with carbapenem resistance was tested using the broth microdilution method (ComASP™ Colistin, Liofilchem, Italy) according to the manufacturer's recommended protocol. The ComASP™ Colistin test comprises seven wells containing lyophilized colistin concentrations that increase twofold (0.25-16 μg/mL). In accordance with the manufacturer's instructions, 10 μl of the bacterial inoculum, adjusted to 0.5 McFarland, was transferred to cation-adjusted Mueller-Hinton broth, which was commercially provided by the manufacturer. Subsequently, 50 μl of the aforementioned broth was distributed into the wells of the microplate. The microplate was then covered with a transparent plastic film and incubated at 37°C for 24 hours, after which it was evaluated.

Investigation of the molecular basis of resistance

Nucleic acid isolation was performed using the Pure Gene DNA Isolation Kit (Thermo Scientific, USA). Molecular detection of *mcr*-1 to *mcr*-8 genes in isolates found resistant to colistin by the broth microdilution method was investigated by multiplex PCR using the Bio-Speedy Colistin Resistance qPCR kit (Bio-Speedy, Turkey) in accordance with the manufacturer's recommendations. The specific primers utilized for the amplification of the *mcr *genes are presented in Table 1.

**Table 1:** Primers used for the detection of mcr genes

Primer Name	Sequence (5ʹ-3ʹ)	Target Gene	Size (bp)
*mcr*-1_205F	TCCAAAATGCCCTACAGACC	*mcr*-1	205
*mcr*-1_205R	GCCACCACAGGCAGTAAAAT		
*mcr*-2_279F	CCTTTTGTGCTGATGGGTTT	*mcr*-2	279
*mcr*-2_279R	ATTTTGGAGCATGGTGGTGT		
*mcr*-3_347F	CTTGCTGAACCAATCCCATT	*mcr*-3	347
*mcr*-3_347R	CCATCGTTCTCCTTCCAAAA		
*mcr*-4_426F	GATCCGAAGCTGTGTTCTG	*mcr*-4	426
*mcr*-4_426R	GCCAGCATTGGTACGCTAGT		
*mcr*-5_522F	GGTTGGCCGAGAAGATAACA	*mcr*-5	522
*mcr*-5_522R	ATGTTGCCAGAAGGTCCAAC		
*mcr*-7_791F	GTCAGTTACGCCATGCTCAA	*mcr*-7	791
*mcr*-7_791R	TTCTTGTCGCAGAACTGTGG		
*mcr*-8_943F	AAACTGAACCCGGTACAACG	*mcr*-8	943
*mcr*-8_943R	GCCATAGCACCTCAACACCT		

*E. coli *NTCC 13846 strain carrying *mcr*-1 gene was used as a positive control strain in the molecular studies. Amplification was performed on a Bio-Rad CFX 96 (Bio-Rad, USA) real-time PCR device. The multiplex PCR protocol for the *mcr-1* to *mcr-8* gene regions is provided in Table 2. The multiplex PCR kit used is for rapid diagnostic purposes and is used to screen genes from *mcr*-1 to *mcr*-8. In case of positive result detection, PCR amplification using standard primers and identification by sequence analysis were planned.

**Table 2:** Multiplex PCR protocol for mcr-1 to mcr-8 genes

Cycle count	Temperature	Duration
1	52°C	5 min
1	95°C	10 sec
40	95°C	1 sec
	55°C	1 sec

Statistical analysis

The statistical analysis of the study was conducted using the IBM SPSS Statistics for Windows, Version 20.0 (released 2011, IBM Corp., Armonk, NY). Descriptive statistics were expressed as number and percentage values. Whether there was a statistically significant difference between meropenem-sensitive and resistant isolates in colistin-resistant strains was investigated using the chi-square test, and a p-value less than 0.05 was considered significant.

## Results

During the study period, a total of 2,646 Enterobacterales isolates were identified among the infectious agents isolated from clinical specimens sent to the laboratory. Of all the isolates identified at the species level by the VITEK 2 automated system and subjected to antibiotic susceptibility testing with MIC value detection, 79 isolates were found to be carbapenem resistant, and there were no repeated samples from the same patient. The carbapenem resistance detected by the automated system in all these samples was confirmed by gradient testing.

According to the results of the colistin broth microdilution test of carbapenem-resistant Enterobacterales strains, colistin resistance was detected in a total of 27 samples and included in the molecular part of the study.

**Figure 1:**
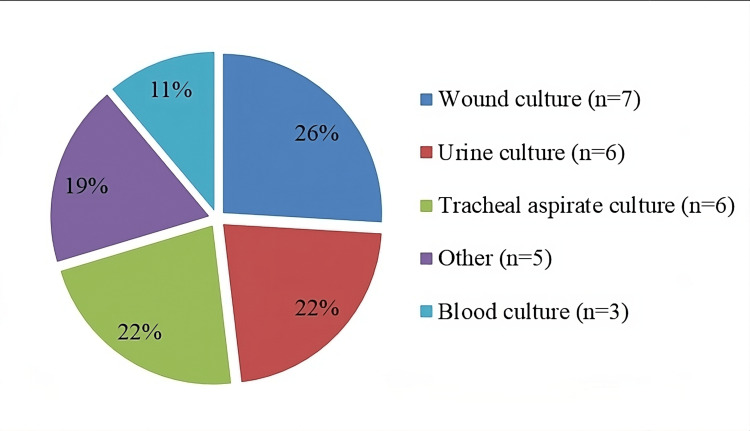
Distribution of isolates with detected colistin resistance according to clinical specimens.

Colistin-resistant strains were most frequently isolated from wound (7, 26%), tracheal aspirate (6, 22.2%), and urine (6, 22.2%) samples. More rarely, they were also isolated from sputum, bronchoalveolar lavage, blood, and sterile body fluids. The distribution of isolates with detected colistin resistant according to clinical specimens is presented in Figure 1. There was no gender difference between the growths; 15 (55.5%) of these bacteria were obtained from male patients and 12 (44.5%) from female patients.

The distribution of isolates included in the study according to the clinics to which they were sent is shown in Table 3. When the samples were evaluated on a clinical basis, the most common isolates were sent from anesthesia intensive care and general surgery services with a rate of 18.5%. It is noteworthy that, as expected, the majority of samples came from intensive care units and all samples belonged to inpatients.

**Table 3:** Distribution of services from which colistin-resistant strains were sent

Clinics	Number of Samples	%
Anesthesiology and Reanimation Intensive Care Unit	5	18.5
General Surgery	5	18.5
Internal Medicine Intensive Care Unit	4	14.8
Neonatal Intensive Care Unit	4	14.8
Pulmonology	3	11.2
Other	6	22.2
Total	27	100

The study revealed that *K. pneumoniae *was the most prevalent pathogen among Enterobacterales isolates exhibiting colistin resistance, with a prevalence of 66.7% and 18 strains. *Proteus mirabilis* was the second most common, with eight strains (29.6%), and *Escherichia coli *had only one strain (3.7%).

The MIC values of colistin for carbapenem-resistant Enterobacterales strains investigated in this study are presented in Table 4. When comparing meropenem-sensitive and resistant isolates in colistin-resistant strains, no significant difference was detected between the two groups (p > 0.05). In our in vitro studies, the MIC50 and MIC90 values for meropenem were found to be >8 µg/ml, whereas for colistin, the MIC50 value was >16 and the MIC90 value was 8 µg/ml. Within the scope of the study, colistin resistance rate was determined as 34.1% in carbapenem-resistant Enterobacterales isolates.

**Table 4:** Colistin MIC values for carbapenem-resistant Enterobacterales strains *R: resistant, MIC: minimum inhibitory concentration

Sample (n=27)	Isolates	Meropenem (Vitek 2)		Colistin (Microdilution Method)	
		MIC (µg/ml)	Comment	MIC (µg/ml)	Comment
1	Proteus mirabilis	>8	R*	>16	R
2	Klebsiella pneumoniae	>8	R	>16	R
3	Proteus mirabilis	>8	R	>16	R
4	Klebsiella pneumoniae	>8	R	>16	R
5	Klebsiella pneumoniae	>8	R	>16	R
6	Klebsiella pneumoniae	>8	R	>16	R
7	Klebsiella pneumoniae	>8	R	8	R
8	Proteus mirabilis	4	R	>16	R
9	Klebsiella pneumoniae	>8	R	4	R
10	Klebsiella pneumoniae	>8	R	4	R
11	Proteus mirabilis	>8	R	>16	R
12	Klebsiella pneumoniae	>8	R	8	R
13	Klebsiella pneumoniae	>8	R	>16	R
14	Proteus mirabilis	>8	R	>16	R
15	Klebsiella pneumoniae	>8	R	8	R
16	Proteus mirabilis	>8	R	>16	R
17	Klebsiella pneumoniae	>8	R	4	R
18	Proteus mirabilis	>8	R	>16	R
19	Klebsiella pneumoniae	8	R	>16	R
20	Klebsiella pneumoniae	>8	R	4	R
21	Klebsiella pneumoniae	>8	R	>16	R
22	Escherichia coli	>8	R	4	R
23	Klebsiella pneumoniae	>8	R	8	R
24	Proteus mirabilis	>8	R	>16	R
25	Klebsiella pneumoniae	>8	R	>16	R
26	Klebsiella pneumoniae	>8	R	>16	R
27	Klebsiella pneumoniae	>8	R	8	R

**Figure 2:**
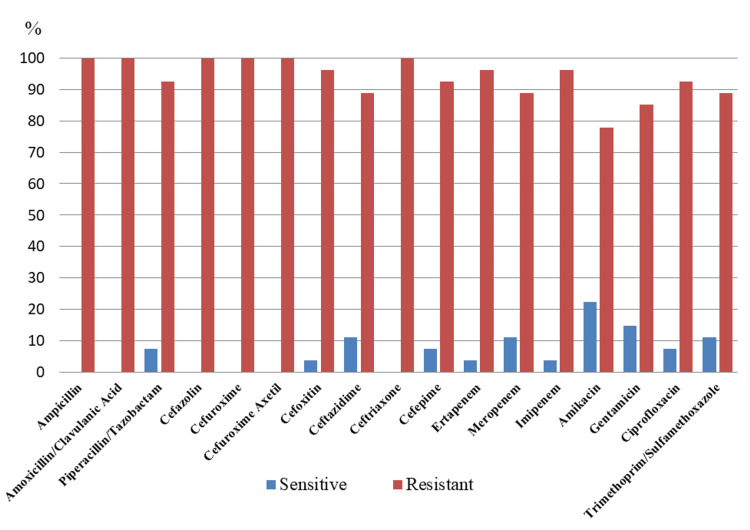
Antimicrobial resistance rates of colistin-resistant isolates

The antibiotic susceptibility profile of colistin-resistant isolates included in the study are presented in Figure 2. In terms of resistance rates, amikacin was the most effective agent with a resistance rate of 77.8%, followed by gentamicin with 85.2%.

**Figure 3:**
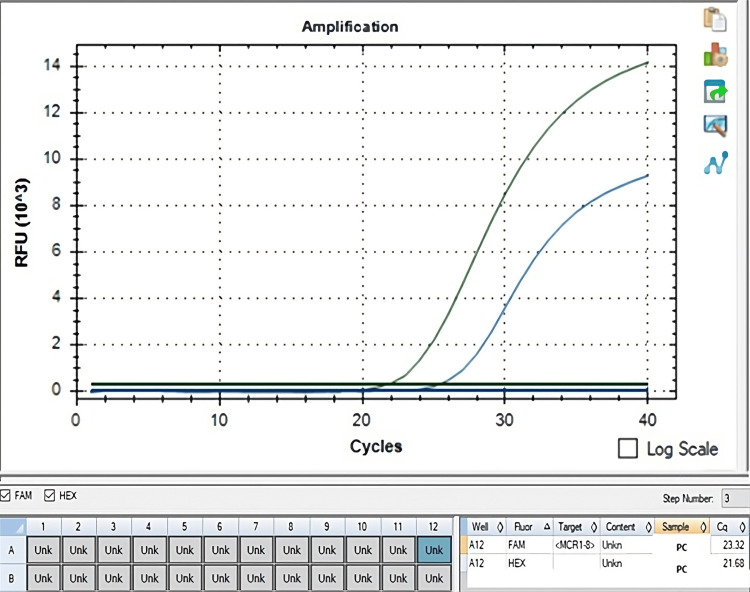
PCR analysis of E. coli NCTC 13846, a positive control strain for mcr-1

The multiplex PCR study conducted to ascertain the prevalence of plasmid-mediated colistin resistance genes in enterobacterales isolates did not yield any positive results for genes *mcr*-1 to *mcr*-8. Figure 3 illustrates the amplifications observed with the positive control in the multiplex qPCR study. In multiplex PCR, data acquisition is performed in the FAM (green/target region) and HEX (blue/internal control) fluorescence channels. The simultaneous amplification curve in the FAM (cycle threshold (ct) = 23.32) and HEX (ct = 21.68) channels in Figure 3 indicates that the sample carries the target *mcr*1-8 gene region.

## Discussion

Given the rapid emergence and dissemination of antibiotic resistance, it is imperative to monitor antibiotic usage and implement appropriate treatment strategies to minimize indiscriminate and incorrect antibiotic use [11]. Colistin, also referred to as polymyxin E, is a polypeptide antibiotic. Its utilization was ceased in the mid-1980s due to neurotoxic and nephrotoxic side effects. However, colistin, an antiquated class of antimicrobials, has resurfaced in clinical settings as a last-resort therapy for infections caused by multidrug-resistant and carbapenem-resistant bacteria. Colistin functions by targeting the outer membrane lipopolysaccharide (LPS) of gram-negative bacteria. Its binding to LPS enhances bacterial membrane permeability, resulting in the leakage of cytoplasmic contents and eventual cell death [12].

Recent escalation in the clinical usage of colistin and the growing demand for it have underscored the significance of colistin susceptibility testing. Studies have illuminated challenges in accurately determining colistin susceptibility. The European Committee on Antimicrobial Susceptibility Testing (EUCAST) advocates for the broth microdilution method using only sulfate salt, sans the addition of surfactants, for ascertaining colistin susceptibility, and discourages the use of disk diffusion and gradient tests. Moreover, the EUCAST recommends employing *E. coli *ATCC 25922 (susceptible) and *mcr-1-*positive *E. coli* NCTC 13846 (resistant) strains for quality control purposes. In our investigation, colistin resistance was assessed using the broth microdilution method in adherence to the EUCAST guidelines. The EUCAST established breakpoints for colistin for Enterobacterales isolates with a susceptible breakpoint of ≤2 mg/liter and a resistant breakpoint of >2 mg/liter [13].

Moreover, the escalating proliferation of resistance within Enterobacterales species presents a substantial concern, limiting therapeutic options. Isolates demonstrating carbapenem resistance exhibit in vitro resistance to all beta-lactam antibiotics and frequently manifest resistance to quinolones as well. Consequently, colistin is employed as a last-resort therapeutic option. Nevertheless, the extensive utilization of colistin in carbapenem-resistant enteric bacteria has precipitated critical levels of colistin resistance in Enterobacterales strains [14]. Colistin resistance can emerge through diverse mechanisms, including chromosomal mutations and plasmid-mediated resistance [4].

Various studies utilizing different reference methods worldwide have reported disparate results. In their study, Rojas et al. investigated colistin susceptibility among patients infected or colonized with *K. pneumoniae *between 2011 and 2014, identifying colistin resistance in 13% of 246 patients. In addition, it was observed that colistin-resistant *K. pneumoniae* infections were associated with high mortality rates [14].

In their study, Özkul Koçak et al. determined colistin resistance in 81 isolates using the microdilution method, finding a resistance rate of 39.5%. Furthermore, the colistin MIC value ranged from <0.0625 to >64 µg/ml using the broth microdilution method [15].

In our study, a total of 27 colistin-resistant strains were identified among 79 carbapenem-resistant Enterobacterales isolates. The most frequently isolated agent in colistin-resistant strains was *K. pneumoniae* at a rate of 66.7%, followed by *P. mirabilis *at 29.6% and *E. coli *at 3.7%. The colistin resistance rate among carbapenem-resistant strains in our study was found to be 30.4%. The colistin MIC value ranged from 4 to >16 µg/ml in strains tested using the broth microdilution method, and our colistin resistance rates were found to be consistent with the literature (Table 4).

Investigating genes responsible for resistance to different antibiotics in bacteria, including plasmid-mediated genes, such as *mcr *genes, is of paramount importance for public health [16]. At present, *mcr* genes have proliferated globally, imperiling the effectiveness of treatments against drug-resistant bacteria unresponsive to conventional antibiotics. The "One Health" paradigm recognizes the interconnectedness of human health with that of animals and the environment, particularly concerning antimicrobial resistance genes. Notably, *mcr* genes pose a significant risk to human health due to their widespread dissemination potential originating from animal breeding, production, international animal trade, and food consumption. Moreover, they pollute our environment through the intricate food chain. Research has elucidated that mcr genes can be sourced from diverse origins, spanning animals, food items, human reservoirs, and environmental habitats, involving a spectrum of pathogens like* E. coli, K. pneumoniae, Salmonella enterica, Cronobacter sakazakii, Raoultella ornithinolytica, Aeromonas,* and *Enterobacter *spp. Predominantly, isolates harboring *mcr *genes belong to the Enterobacterales isolates, prevalent in both animal and human populations [12]. In a retrospective analysis conducted by Shen and colleagues, it was revealed that the *mcr*-1 gene was present in *E. coli *strains derived from poultry in China as early as the 1980s. This implies a plausible correlation between the inception of *mcr* genes and the utilization of colistin within the poultry sector. The earliest detection of the *mcr* gene (specifically *mcr*-1) in humans occurred in a *Shigella sonnei *strain isolated from a pediatric patient hospitalized in Vietnam in 2008 [17,18]. Out of the 10 known variants of *mcr *genes discovered thus far (*mcr*-1 through *mcr*-10), *mcr*-1 is recognized as the predominant and extensively spread lineage, with *mcr*-3 following closely behind [19]. Since 2010, there has been a notable rise in *mcr-*positive isolates identified in both animal and human populations. While all *mcr* genes have been detected in animal specimens, *mcr*-6 and *mcr*-7 have yet to be reported in human samples [12].

The dissemination of *mcr* genes is possible across bacterial strains derived from animals, humans, and environmental sources. On the other hand, since only human samples were studied, positivity may not be detected at the rates stated in the literature. Multiple bacterial species might possess genetic elements spanning from *mcr*-1 to *mcr*-9, facilitating the transfer of these genes across diverse ecosystems, including terrestrial, aquatic, wildlife, and botanical habitats, via horizontal gene transfer mechanisms [20].

Numerous studies have introduced diverse methodologies for identifying the *mcr* gene family, encompassing standard PCR, multiplex PCR spanning from *mcr*-1 to *mcr*-9, SYBR green fluorescent qPCR, TaqMan fluorescent qPCR, and alternative approaches [21]. Loop-mediated isothermal amplification (LAMP) assays are accessible for swift identification of genes ranging from *mcr*-1 to *mcr*-5 in colistin-resistant bacteria. Notably, LAMP demonstrates heightened sensitivity and specificity relative to conventional PCR. Nevertheless, owing to the heterogeneity of *mcr *genes, a solitary LAMP assay might fail to capture all intended targets, thus falling short for nucleic acid detection. Instances involving specimens harboring multiple documented mcr genes may challenge the sensitivity and specificity of multiplex LAMP tests [22]. Hu et al. devised a Quad-PCR technique for expedited and dependable detection of prevalent *mcr*-1,* mcr*-3, *mcr*-8, and *mcr*-10 genes within clinical samples [23].

A recent investigation has introduced a swift, efficient, and precise technique by merging lateral flow dipstick (LFD) identification with recombinase polymerase amplification (RPA). Nevertheless, this approach exclusively targets the* mcr*-1 gene. However, research suggests that *mcr*-9 and *mcr*-10 are progressively surfacing in clinical cohorts and are proliferating globally [24]. These methodologies only capture a subset of the *mcr *genes, and detecting certain variants entails prolonged efforts. Hence, the development of swift detection methods encompassing the entire spectrum of reported *mcr* gene variants is imperative. In our study, we utilized a multiplex PCR kit to explore the presence of plasmid-borne gene sequences spanning from *mcr*-1 to *mcr*-8.

In their 2018 investigation conducted in Egypt, Zaki MES et al. included 50 Enterobacterales strains and examined the presence of the *mcr*-1 and *mcr*-2 genes in isolates exhibiting colistin resistance MIC >2 mg/L. Utilizing PCR analysis, they identified the *mcr*-1 gene in one* E. coli *and one *K. pneumoniae* strain [25].

Dalmolin et al., in their 2018 research conducted in Brazil, focused on studying the *KPC*-2 gene region in carbapenem-resistant strains. In addition, they investigated the plasmid mediated *mcr*-1 gene region associated with colistin resistance. They detected the *mcr*-1 gene in a *K. pneumoniae* strain. These findings underscore the potential emergence of *K. pneumoniae* isolates harboring both *mcr*-1 and *KPC*-2 genes as a significant challenge to antimicrobial therapy [26].

In contrast to* E. coli*, where mcr genes and variants have been identified with increasing frequency in isolates from human, animal, and environmental samples worldwide, *K. pneumoniae* has a lower frequency of *mcr*. Chen et al. screened genes from *mcr*-1 to *mcr*-8 in 47 colistin-resistant *K. pneumonia* isolates from 2010, 2012, 2014, and 2016 collected under the Taiwan Antimicrobial Resistance Surveillance Program and found no positivity. However, in their retrospective study, they identified *mcr*-1 in two isolates, *mcr*-3 in one, and *mcr*-8 in three among 24 *K. pneumoniae* isolates, from 2018 collection with colistin MIC values >2 mg/L. Subsequent Sanger sequencing revealed these variants as* mcr*-1.1, *mcr*-3.5, and *mcr*-8.2, respectively. Chen et al. documented that strains containing *mcr* genes were sourced from various clinical specimens collected from elderly inpatients across different medical facilities. They observed that while these isolates demonstrated resistance to multiple antibiotics, they retained susceptibility to carbapenems and amikacin. They indicated that plasmids carrying either *mcr*-1, *mcr*-3, or *mcr*-8 have spread to clinical *K. pneumoniae* isolates from different parts of Taiwan, requiring continuous monitoring due to their transferability, potential spread, and association with multiple antimicrobial resistance genes [27].

In our study, the presence of plasmid mediated *mcr*-1 to *mcr*-8 gene regions was investigated in 27 colistin-resistant Enterobacterales strains isolated from various clinical specimens. While the *mcr*-1 gene region was detected in the positive control isolate by real-time PCR, none of the *mcr*-1 to *mcr*-8 gene regions were identified in our study where we investigated the presence of plasmid-mediated genes using a multiplex PCR kit.

Limitations

The most significant limitation of our study is the low number of colistin-resistant Enterobacterales strains. The reduction in the number and diversity of inpatients during the pandemic period, when the study was planned, negatively impacted the agent profile isolated during that period. 

Another limitation of the study is that although the successful detection of the positive control carrying the *mcr*-1 gene with the multiplex PCR kit used indicates that there is no study-related problem, demonstrating that the positive controls of different *mcr* genes also work would enhance the meaningfulness of the results. While the *mcr*-1 gene region was detected in the positive control isolate by real-time PCR, none of the *mcr*-1 to *mcr*-8 gene regions were identified in our study where we investigated the presence of plasmid-mediated genes using a multiplex PCR kit. As a consequence of the failure to detect positivity, it was not possible to proceed with the planned advanced molecular examinations, which constituted another key element of the study.

## Conclusions

The development of resistance to colistin, which is used as a last resort, is undesirable where carbapenem resistance among gram negative bacteria is increasingly prevalent. The multiplex PCR method can be used to provide information on the underlying resistance mechanism and to reveal the presence of potentially known *mcr *genes in isolates that are resistant or borderline susceptible after MIC determination by standard broth microdilution. Although the multiplex PCR method cannot be used to determine which gene is specifically present, it provides valuable information for research and epidemiologic surveillance. The multiplex PCR results will guide the selection of isolates for further studies based on genomic epidemiology, focusing on the characterization of *mcr *genes, their mobilization mechanisms and the plasmids they harbor.

In summary, the multiplex PCR method, which rapidly detects the *mcr-*1-8 genes present in Enterobacteriaceae, revealed the absence of these genes in clinical isolates exhibiting colistin resistance. At the current stage, it can be reasonably concluded that the emergence of colistin resistance in our region is not attributable to *mcr-*1-8 genes. It is also necessary to investigate non-*mcr* mechanisms in colistin-resistant isolates in our region.
